# Expression of connexin genes in the human retina

**DOI:** 10.1186/1471-2415-10-27

**Published:** 2010-10-27

**Authors:** Goran Söhl, Antonia Joussen, Norbert Kociok, Klaus Willecke

**Affiliations:** 1 Institut für Genetik der Universität Bonn, Römerstr. 164, 53117 Bonn, Germany; 2 Zentrum für Augenheilkunde der Universität Köln, Abteilung für Netzhaut und Glaskörperchirurgie, Kerpener Str. 62, 50924 Köln, Germany; 3 Martinus Gymnasium Linz, Martinusstraße 1, 53545 Linz am Rhein, Germany; 4 Klinik für Augenheilkunde der Charité - Universitätsmedizin Berlin Campus Benjamin Franklin, Hindenburgdamm 30, 12200 Berlin, Germany; 5 Augenklinik des Universitätsklinikums Düsseldorf, Moorenstr. 5, 40225 Düsseldorf, Germany; 6 LIMES Institut, Universität Bonn, Carl-Troll-Str. 31, 53115 Bonn, Germany

## Abstract

**Background:**

Gap junction channels allow direct metabolically and electrical coupling between adjacent cells in various mammalian tissues. Each channel is composed of 12 protein subunits, termed connexins (Cx). In the mouse retina, Cx43 could be localized mostly between astroglial cells whereas expression of Cx36, Cx45 and Cx57 genes has been detected in different neuronal subtypes. In the human retina, however, the expression pattern of connexin genes is largely unknown.

**Methods:**

Northern blot hybridizations, RT-PCR as well as immunofluorescence analyses helped to explore at least partially the expression pattern of the following human connexin genes GJD2 (hCx36), GJC1 (hCx45), GJA9 (hCx59) and GJA10 (hCx62) in the human retina.

**Results:**

Here we report that Northern blot hybridization signals of the orthologuous hCx36 and hCx45 were found in human retinal RNA. Immunofluorescence signals for both connexins could be located in both inner and outer plexiform layer (IPL, OPL). Expression of a third connexin gene denoted as GJA10 (Cx62) was also detected after Northern blot hybridization in the human retina. Interestingly, its gene structure is similar to that of Gja10 (mCx57) being expressed in mouse horizontal cells. RT-PCR analysis suggested that an additional exon of about 25 kb further downstream, coding for 12 amino acid residues, is spliced to the nearly complete reading frame on exon2 of GJA10 (Cx62). Cx59 mRNA, however, with high sequence identity to zebrafish Cx55.5 was only weakly detectable by RT-PCR in cDNA of human retina.

**Conclusion:**

In contrast to the neuron-expressed connexin genes Gjd2 coding for mCx36, Gjc1 coding for mCx45 and Gja10 coding for mCx57 in the mouse, a subset of 4 connexin genes, including the unique GJA9 (Cx59) and GJA10 (Cx62), could be detected at least as transcript isoforms in the human retina. First immunofluorescence analyses revealed a staining pattern of hCx36 and hCx45 expression both in the IPL and OPL, partially reminiscent to that in the mouse, although additional post-mortem material is needed to further explore their sublamina-specific distribution. Appropriate antibodies against Cx59 and Cx62 protein will clarify expression of these proteins in future studies.

## Methods

Gap junction channels allow direct metabolic and electrical communication between neighboring cells. They are composed of two hemi-channels also denoted as connexons, each constisting of six connexin protein subunits [1]. Gap Junctions are widely distributed among mammals and known to be expressed in a spatio-temporal fashion [2] i.e. in both the retina as well as in the CNS [3-5].

In the vertebrate retina, gap junctions are found between nearly all cell types [6]. Cone pedicles are coupled to each other as well as to rod spherules [7]. Only the same subtypes of horizontal cells are coupled between their dendrites, somata and axons [8-11]. Gap junctional coupling of bipolar cells is present between axons or dendrites of either the same or different subtypes [12]. Amacrine cell coupling is still largely unexplored but their homologous dendro-dendritic gap junctions (mostly between AII-AII cells, see [13]) can be readily distinguished from the heterologous junctions connecting them to ON-cone bipolar cells [14-16]. Finally, both homologuous as well as heterologuous coupling is known for ganglion and amacrine cells [17-19].

To study the cell-type specific expression profile of different connexins in the retina, transgenic mouse models have been generated [5]. Among the nearly 20 different connexin genes discovered in the mouse and human genome [20], Cx36 protein expression could be localized between AII-amacrine cells [21], in cone pedicles and OFF-cone bipolar cells [22] and between dendrites of the α-type ganglion cells [23,24]. Cx45 protein detected in the IPL and OPL [25], was later localized adjacent to Cx36 in ON-cone bipolar cells, presumably contributing to heterologuous gap junctions connecting AII-amacrine cells to ON-cone bipolar cells [26]. Furthermore, Cx45 was demonstrated between bistratified ganglion cells [27]. Targeted deletion of Cx36 in the mouse retina subsequently led to visual transmission defects including a reduction of the b-wave [28] and the elimination of rod-mediated, ON-center responses at the ganglion cell level [29]. Subsequently, the ablation of the Cx45 coding region also resulted in a reduction of the b-wave similar to what was observed after ablation of the Cx36 protein [28]. Expression of the Cx57 gene was discovered in mouse retinal horizontal cells after replacing the Cx57 coding region by the lacZ reporter gene [30]. To date, other connexin isoforms (Cx31, Cx32 and Cx40) can be largely excluded from retinal neurons [31] or are known to occur in astrocytes of the mouse retina [25].

However, expression of connexin isoforms in the primate or human retina is still largely unexplored. Primate cones were apparently interconnected by gap junctions that might cause a modest decrease in human color discrimination coinciding with an increase in luminance discrimination [32]. Is the expression pattern of connexin genes in the human retina similar to the connexin expression pattern in mouse retina?

Three years ago the nomenclature of the mouse (http://www.informatics.jax.org search on "connexin" to go the complete list) and human (http://genenames.org/genefamily/gj.php) connexin genes has been extended in parallel. According to this nomenclature the human genes coding for hCx36 and hCx45 are abbreviated as GJD2 and GJC1, respectively, whereas hCx59 and hCx62 are abbreviated as GJA9 and GJA10, respectively.

Here we have investigated the expression profile of hCx36 and hCx45, known as the putative human orthologs to mCx36 and mCx45, in human retina. Their transcripts were detected after Northern blot hybridization as well as immunopositive signals suggesting the presence of hCx36 and hCx45 proteins in both the inner and outer plexiform layers. Additionally, mRNA of hCx62, the putative human ortholog of mCx57 (Gja10), was found after Northern blot hybridization. HCx59 (GJA9), having no ortholog in the mouse genome [20,33], shows a high phylogenetic sequence relationship to zebrafish Cx52.6 [34], zebrafish Cx55.5 [35] but much less to mCx57 and porcine Cx60 [55]. However, in contrast the hCx62, hCx59 transcripts could not be detected after Northern blot hybridization, presumably due to their very low abundance. Therefore, we also tested expression of hCx59 and hCx62 by RT-PCR and found specific amplicons in cDNA synthesized from total human retinal RNA. Consequently, hCx62 is likely to be spliced in accordance with its mouse ortholog mCx57 [30], i.e. splicing of a third exon to the C-terminal leads to replacement of the last 63 amino acid residues by the same 12 amino acid residues [NMLLELSSIMKK] discovered in the spliced mouse Cx57 cDNA. The future availability of specific antibodies against hCx59 and hCx62 proteins should elucidate the presence of these proteins in human retina.

## Methods

### Sequence analysis

We have used the "ClustralW" protein sequence alignment program of the HUSAR/EMBL/Heidelberg platform to establish a multiple sequence alignment file [.msf] of the connexins zebrafish Cx52.6 (acc. no. **AAM46775**), zebrafish Cx55.5 (acc. no. **AAG24878**), mCx57 (acc. no. **CAB40358**), porcine Cx60 [55], hCx59 (acc. no. **AAG09406**), hCx62 (acc. no. **CAC93847**). Using this file, the program "Phylip2Tree" calculated the phylogenetic relations between these connexins.

Alignments of mouse the Cx57 sequence to the human Cx59 and Cx62 sequences were done by using the Human-Mouse alignment browser with the "BLASTZ" algorithms. The tracks obtained display blastz alignments of the Feb. 2003 mouse draft assembly to the human genome filters to show only the best alignment for any given region of the human genome. The tracks indicate a graphical score of the alignment. These alignments were contributed by Scott Schwartz from the Penn State Bioinformatics Group. The best in genome filtering is done by UCSC's axtBest program. The methods and the filter implementation were described in detail [36].

### Tissue preparation

Adult 3- month- old male mice (C57BL/6) were deeply anesthetized with 2 ml Forene^® ^airway application for 5 min according to the instructions. Then the mice were decapitated and tissues were quickly frozen in liquid nitrogen. All animal experiments were performed according to the guidelines of the German law for the welfare of animals.

Human eyes were obtained from a female patient who died at an age of 93 years of colon cancer. Her relatives agreed to the organ donation after death and the dissection was performed in compliance with the Helsinki declaration.

Tissues from a human eye were prepared after 16 h from a post-mortem biopsy cooled at 4°C. Retina, lens, iris diaphragm and optical nerve stump was prepared and directly frozen in liquid nitrogen or Tissue Tek^®^.

### Northern blot analysis

Total RNA from the different mouse and human tissues was prepared with TRIzol^®^-reagent (GibcoBRL) according to the manufacturer. RNA (20 μg) was electrophoresed and transferred to HybondN nylon membrane (Amersham International, Amersham, Bucks, UK) by capillary diffusion in 20 × SSC [37]. Northern membrane was probed by using corresponding hybridization fragments of mouse Cx36, mouse Cx45 [38] and human Cx59 (1.5 kb) as well as human Cx62 (1.6 kb) genomic DNA (this study). Subsequently, the amounts of total RNA on the Northern blot were standardized by hybridization of to a probe of glyceraldehydephosphate dehydrogenase (GAPDH) [39]. Probes were ^32^P-labelled, using the random primed labelling method (Amersham, Amersham, Bucks, UK) to a specific activity of 0.5 to 1.0 × 10^9 ^cpm/μg DNA and added to fresh QuikHyb^® ^hybridization solution (Stratagene, La Jolla, CA, USA) at 1.4 × 10^6 ^cpm/ml. Hybridization at high stringency was carried out for 1 h at 68°C. The filters were finally washed for 30 min in 0.1 × SSC/0.1% SDS at 60°C and exposed to XAR X-ray film (Eastman Kodak, Rochester, NY, USA) with intensifying screen at -70°C for three weeks. The amounts of total RNA on the Northern blot were roughly standardized by determination of the intensities of the ethidium bromide stained 18S- and 28S rRNA.

### RT-PCR analysis

Reverse transcription of total RNA from mouse and human tissues was performed according to [40]. Aliquots of the transcribed cDNA (1/25 from tissue and cells [approximately 0.1 ng]) were amplified using corresponding primer combinations listed in Table 1. Reaction mixtures (50 μl) contained 20 mM Tris-HCl (pH 8.4), 250 μM dNTPs, 1.25 mM MgCl_2_, 50 mM KCl, 2 μM of each primer and 1 unit Taq DNA-polymerase (Promega, Madison, Wisconsin, USA). PCR was carried out for 40 cycles using a PTC-200 Thermal Cycler (MJ Research, Watertown, MA, USA) with the following program: first denaturing step at 94°C for 3 min, denaturing at 94°C for 1 min, annealing at 55°C for 1 min, elongation at 72°C for 2 min and final elongation for 7 min. After gel electrophoresis in an 1% agarose gel the ethidium bromide stained fragments were documented in [37]. Fragments of interest were excised from the gel, purified by using the QiaQuick^® ^purification procedure for PCR-fragments (Qiagen, Hilden, Germany) and finally subcloned into the pGEM-T_easy_^® ^vector system suited for cloning PCR-fragments (Promega) which were commercially sequenced by AGOWA, Berlin, Germany.

**Table 1:** Primer sequences used for human Cx59 (GJA9) and Cx62 (GJA10) RT-PCR analyses

primer	sequence	
hCx59 USP1	5'- atg-ggg-gac-tgg-aat-ctc-ctt-g	(22 nt)

hCx59 USP2	5'- ttg-gag-caa-gag-ctt-tgt-cag	(21 nt)

hCx59 DSP1	5'- ttt-ggc-tgg-aat-aca-gaa-gat-g	(22 nt)

hCx59 DSP2	5'- tta-gat-ctg-aag-atc-tgt-ggg	(21 nt)

		

hCx62 USP1	5'- atg-ggg-gac-tgg-aac-tta-ttg-g	(22 nt)

hCx62 USP2	5'- gga-gca-gca-aag-aat-aga-tag-g	(22 nt)

hCx62 DSP1	5'- ctt-caa-gtt-gcc-ttg-gct-gtg	(21 nt)

hCx62 DSP2	5'- tga-att-gaa-ttt-aac-tga-atg-tac	(21 nt)

hCx62 DSP3	5'- tta-ttt-ttt-cat-aat-aga-tga-aag-ttc	(27 nt)

hCx62 DSP4	5'- tta-agg-ctc-ttt-tct-tac-aaa-aat-aag	(27 nt)

### Immunofluorescence analysis

Cryosections (12 μm) of retinae and control tissues from the human eye were fixed in absolute ethanol (-20°C) for 10 min, washed in PBS for 5 min and preincubated for 30 min in blocking reagent (PBS containing 4% BSA and 0.1% Triton X-100). For detection of Cx36 and Cx45, slides were incubated for 1 hour with appropriate dillutions of affinity purified connexin antibodies: polyclonal rabbit-anti mouse Cx36 [Zymed 36-4600; lot-no. 30979684-0905] and polyclonal goat-anti mouse Cx45 [Chemicon AB1748; lot-no. 19070139]. After 3 washes in PBS, samples were stained for 1 hour with the corresponding Cy3- or Alexa488-conjugated secondary antibodies: Cy3-goat anti-rabbit IgG [Dianova; lot-no. 111-165-114] and Alexa488-donkey anti-goat [MoBiTec; lot-no. A-11055]. As negative control, specimen was incubated in blocking solution only. After incubation, slides were washed in PBS and incubated with DAPI (1:1000) in PBS for 5 min. After a short wash step in demineralized water, slides were mounted with Permaflour. Fluorescence signals were recorded by using either a Zeiss Axiophot photomicroscope equipped with a 63× objective and appropriate filters or a Leica TCS confocal microscope. Images were analyzed and prepared for publication by using Adobe Photoshop 6.0.

The above mentioned antibodies against Cx36 and Cx45 proteins have been applied to cyosections of Cx36 and Cx45 wild type versus knock-out mice [25,31]. The punctated immunofluorescence staining pattern typical for connexin proteins was not detectable in retinal sections of the corresponding knock-out mice, whereas the large and bright dots remained and thus were regarded as unspecific staining artifacts.

## Results

### Phylogenetic relationship between mCx57 (Gja10), hCx59 (GJA9) and hCx62 (GJA10)

**Figure 1:**
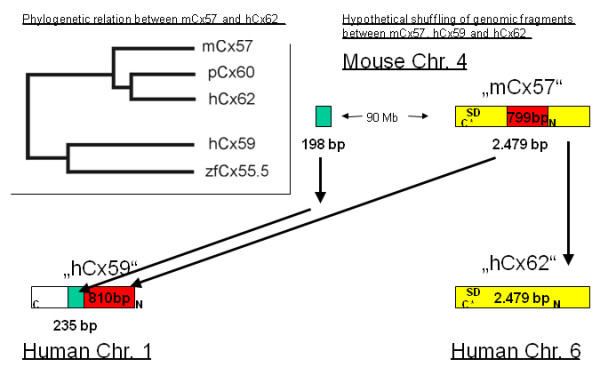
**Phylogenetic tree deduced from a multiple protein sequence alignment revealed the phylogenetical relationship of mouse Cx57, porcine Cx60 and human Cx62**. Instead, human Cx59 (GJA9) seems much more related to both zebrafish connexins Cx55.5 and Cx52.6. Hypothetical shuffling of genomic fragments could occur between mCx57, hCx59 and hCx62. Human-mouse alignments with BLASTZ indicate that human Cx62 (GJA10) (Chr. 6) might be the orthologuous connexin gene to mouse Cx57 (Gja10) (Chr. 4). N; N-terminus, C*; C-terminus * indicates that this stop codon is not used. SD; splice donor site.

Deduced from comparisons of connexin gene pairs, human Cx62 (GJA10) is most likely the orthologuous connexin gene to mouse Cx57 (Gja10) (82% nucleotide identity; 78% amino acid identity; see [20]. A direct comparison between mouse Cx57 (Gja10) and human Cx59 (GJA9) instead yielded much lower sequence identities, suggesting that the Cx59 (GJA9) gene has no mouse ortholog. Results from a phylogenetic alignment, however, indicated that hCx59 (GJA9) might belong to a group of connexin open reading frames (ORFs) of relatively high molecular weight having its closest sequence relationship to zebrafish Cx55.5, with which it was tightly clustered. Both connexin genes are further aligned to a subgroup containing mCx57 (Gja10), porcine Cx60 [55] and hCx62 (GJA10) (dendrogram in Figure 1; accession numbers in Methods).

Closer examination unraveled sequence peculiarities between mCx57 (Gja10), hCx59 (GJA9) and hCx62 (GJA10). A schematic drawing in figure 1 illustrates an uninterrupted sequence of high similarity (yellow) between mCx57 (Gja10) and hCx62 (GJA10) that underscores their direct phylogenetic relationship. Most interestingly, some "islets" of similarity between mCx57 (Gja10) and hCx59 (GJA9) (green and red) indicate also their at least partial relationship. In detail: a mouse sequence of about 2479 bp on **Chr. 4 position contig. [from 32.418.233 to 32.420.711]**was readily aligned with 75.8% sequence identity to a human sequence of about 2529 bp on **Chr. 6 position contig. [from 90.553.469 to 90.555.997]**. However, an internal segment of 799 bp in that mouse sequence also aligned with 65.4% sequence identity to a human sequence of 810 bp on **Chr. 1 position contig. [from 38.804.554 to 38.805.363]**. Moreover, a small sequence of 198 bp approx. 90 Mb upstream of the mentioned mouse sequence **Chr. 4 position contig. [from 121.921.142 to 121.921.339]**was aligned with 68.5% sequence identity to a human sequence of 235 bp **Chr. 1 position contig. [38.804.122 to 38.804.356],**that is located adjacent to the 810 bp sequence. The *Ensembl *gene report of human Cx59 (**Gene ID: ENSG00000131233**) predicts its reading frame on **Chr. 1 position contig. [38.803.761 to 38.805.308]**comprising 1548 bp or 515 aa's. As a rough estimation, about 1186 bp coding for 395 amino acid residues aa's (~55%) of the predicted hCx59 N-terminal coding region can be regarded as similar to mouse Cx57 (Gja10).

### Sequence similarities between mCx57 and hCx62 transcript isoforms

**Figure 2:**
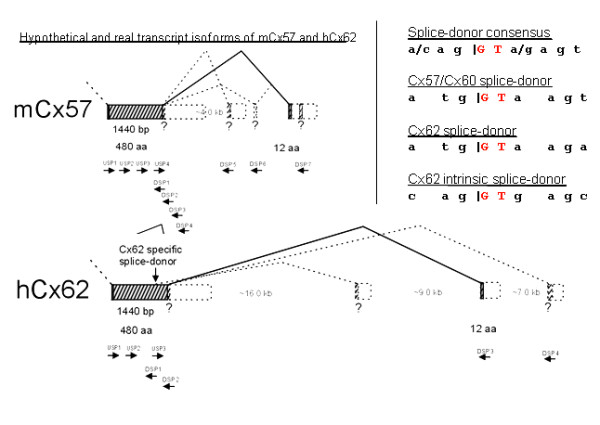
**Hypothetical and real transcript isoforms of hCx62. Schematic drawing of the hCx62 connexin gene (GJA10)**. The gray hatched boxes represent the coding region of hCx62 which is linked by the roof-like black line. The first exon contains 1440 nucleotides coding for 480 amino acid residues and the second exon comprises at least the 36 nucleotides coding for the C-terminal amino acid residues. Both exons are flanked by an intron of about 25 kb. The dashed boxes and lines only represent hypothetical splice isoforms which could not be proven by RT-PCR analyses. The arrows indicate the primers used for RT-PCR analyses. USP; upstream-primer, DSP; downstream-primer. The sequences of the splice donor sites are listed separately.

In a previous study, the C-terminal end of mCx57 (12aa) was demonstrated to be encoded on a putative third exon approx. 9 kb further downstream of the major coding region (480 amino acid residues) that is functionally spliced after transcription [30]. RT-PCR primer combinations between upstream primer (USP) 1 to 4 and downstream primer (DSP) 1 to 2 confirmed the uninterrupted transcription of the major part of the mCx57 coding region on exon2 (data not shown). However, after application of downstream primer DSP3, -4, -5 and -6, the corresponding RT-PCR amplicons failed to emerge in each case (data not shown), thus indicating that neither the C-terminus (25 amino acid residues) is encoded on exon2 (as predicted by [41]), nor that the hypothetical transcript isoforms, deduced from data base predictions, are transcribed in figure 2. Only downstream primer DSP7 yielded an amplicon that was cloned and sequenced [30]. The position of primer DSP7 further downstream of a fourth predicted small reading frame and splice acceptor site concomitantly excluded their usage but instead confirmed the transcription of a small reading frame (36 nucleotides; 12 amino acid residues) and its upstream located splice acceptor site (figure 2).

In order to access the splice pattern of hCx62 transcript isoforms, results obtained from the Gja10 gene structure coding for mCx57 have been extrapolated to the GJA10 gene coding for hCx62. Although a bio-informatical sequences analyses predicted an uninterrupted hCx62 reading frame of about 1629 base pairs (543 amino acid residues) on exon2, a putative splice donor-site, similar to that of mCx57 (figure 2) was found in the C-terminal part of the hCx62 reading frame, thus implying that 63 amino acid residues of the following downstream sequence might remain untranslated. Moreover, a second putative splice-donor site was found upstream only in the hCx62 reading frame but not in the hCx59 and zebrafish Cx55.5 coding region. An alignment of the small mCx57 exon3 sequence to the human genomic sequence of hCx62 resulted in a nearly perfect match approx. 25 kb further downstream of hCx62 exon2. At this position, the same 12 amino acid residues [NMLLELSSIMKK] expressed in mCx57 are encoded, coinciding with a proper splice acceptor site (figure 2). Therefore, different primer combinations for RT-PCR analyses have been selected for detection of putatively unspliced hCx62 transcript isoforms (USP1 to 3 applied with DSP1 or DSP2) or putatively spliced hCx62 transcript isoforms (USP1 to 3 applied with DSP3 or DSP4; see table 1 and figure 2).

**Figure 3:**
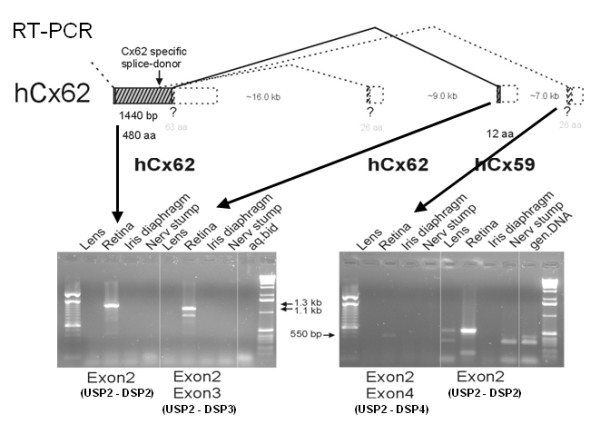
**RT-PCR analyses of hCx59 and hCx62 RNAs in different tissues of the human eye**. Both the hCx62-specific primer combination USP2 - DSP2 (within the first exon) and the primer combination USP2 - DSP3 (intron spanning) yielded amplicons of about 1.3 kb and 1.1 kb, respectively, only with cDNA from retina. The intron-spanning primer combination USP2-DSP4 even failed to yield a signal with retina cDNA. The amplicon of 550 bp implies that hCx59 seemed to be expressed in retina and also very faintly in lens.

RT-PCR analyses revealed the presence of hCx62 as well as hCx59 transcripts only in cDNA from human retina RNA but not in cDNA from human lens, iris diaphragm, and nerve stump RNA (figure 3). In human retinal cDNA, the unspliced form and the spliced hCx62 transcript isoform, containing exon3 approx. 25 kb downstream of exon2, were detected. However, no amplicon corresponding to the expression of a putative hCx62 transcript isoform containing a third exon about 32 kb downstream of exon 2 (figure 3) was found. A very weak amplicon reflecting hCx59 (GJA9) expression in human lens cDNA is likely due to RNA contamination during tissue preparation (figure 3).

**Figure 4:**
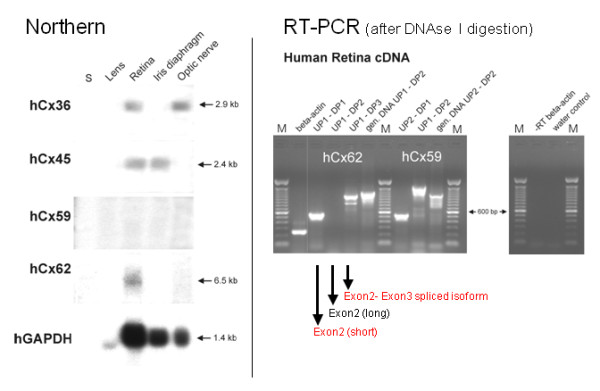
**Northern blot hybridization of total RNA from 4 different tissues of the human eye**. No band, indicating hCx59 expression, was detected even after three weeks of exposure, whereas rehybridization to a probe coding for the human glyceraldehydephosphate dehydrogenase [GAPDH] [39] led to a specific hybridization signal of about 1.4 kb. Hybridization signals for hCx62 were detected in retina and the optical nerve, whereas hybridization signals specific for hCx45 were seen also in retina and the iris diaphragm after two weeks of exposure. Interestingly, a faint signal at 6.5 kb might indicate expression of hCx62 also in the human retina. S; RNA-Ladder (Gibco-BRL). RT-PCR analyses of the expression of hCx59 and hCx62 in human retina cDNA after DNAseI digestion. The spliced 243 bp amplicon but not the 330 bp amplicon of the beta-actin gene demonstrates the absence of genomic DNA from the human retina cDNA pool. Primer combinations USP1-DSP1 and the intron-spanning USP1-DSP3 yielded fragments of the expected size. Primer combination USP1-DSP2 failed to give any signal with cDNA from human retina but instead with human genomic DNA. Different primer combinations for hCx59 yielded amplicons of the expected size (USP2-DSP1; 550 bp and USP1-DSP2; 1.5 kb) when probed with cDNA from human retina. An amplicon of about 1.1 kb was found after applying human genomic DNA. No signals after beta-actin RT-PCR and the empty water control indicate the absence of genomic DNA from the probes.

Thus, selective RT-PCR reactions were repeated after digestion of total RNA from human retina with DNAse I, in order to exclude even residual traces of genomic DNA (see in figure 4). Interestingly, only primer combinations USP1-DSP1 generated a short amplicon of hCx62 exon2 and primer combination USP1-DSP3 confirmed again the presence of the spliced hCx62 isoform containing exon2 and 3. Amplification of the extended exon2 (primer USP1-DSP2), however, failed after DNAse I digestion (figure 4), suggesting the previous amplification product (figure 3) was likely due to contamination with genomic DNA. Thus, expression of the hCx62 as an uninterrupted coding region on exon2 (also including 63 amino acid residues downstream of the splice donor site) in human retinal cDNA could not be confirmed. Expression of human Cx59, however, was still detected after DNAse I digestion and application of two different primer combinations (USP1-DSP2 and USP2-DSP1), underscored that hCx59 is transcribed, at least in traces, in human retina.

### Abundance of connexin transcription in the human retina

Among the different connexins shown to be transcribed and translated in the mouse and rat retina are Cx36, Cx43, Cx45 and Cx57 [25,30,31]. Several studies indicate that mCx43 is functionally expressed in retinal astrocytes [31], while mCx36, mCx45 and mCx57 protein establish functional gap junction channels between various neuronal subclasses of the mouse [5]. Here we have explored whether the human orthologs of the denoted neuronal mouse connexin genes are also expressed in human retina. Hybridization signals of appropriate sizes for hCx36, hCx45, hCx62, but not for hCx59 transcripts could be readily detected after Northern blot hybridization of human retina RNA (figure 4). Additionally, a signal for hCx36 emerged in the RNA sample extracted from human optical nerve and a signal for hCx45 was seen in iris diaphragm (figure 4). Even after longer x-ray exposure time no hybridization signal indicating expression of hCx59 could be detected. Furthermore, none of the four connexins tested was found to be expressed in human lens RNA.

### Immunofluorescence analyses of hCx36 and hCx45 protein expression in the human retina

**Figure 5:**
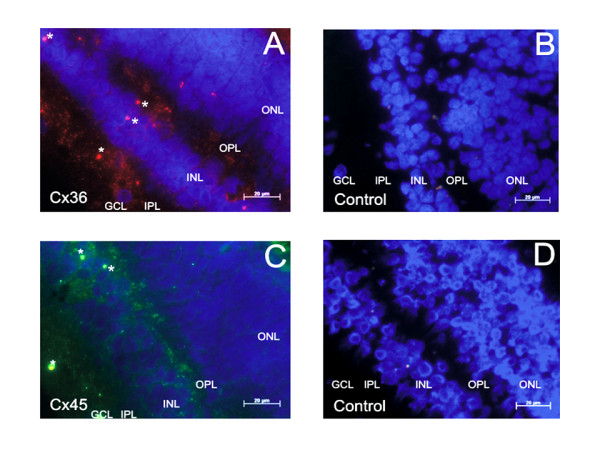
**Immunofluorescence study of Cx36 and Cx45 expression in the human retina**. **A) **An array of red Cx36-immunosignals was detected in the inner plexiform layer (IPL) extending to the ganglion cell layer (GCL) and also in the outer plexiform layer (OPL). **B) **Retinal cryosections after application of only the secondary *Cy3-*antibodies served as control for the specificity of the Cx36 antibodies used. **C) **The Cx45 immunostaining (green) was very faint and the punctate pattern was prominently seen in the outer plexiform layer (OPL) but less obvious in the IPL and GCL. **D) **Retinal cryosections after application of only the secondary *Alexa488-*antibodies served as control for the specificity of the Cx36-antibodies used. All nuclei were stained blue with DAPI. Unusual large staining signals, exemplarily marked by asterisks, were considered as unspecific staining (see Methods). Scale bar: 20 μm.

Due to high protein sequence identities between mouse Cx36 and Cx45 protein and their human counterparts (both at about 98%; [20]), we used antibodies directed against mouse Cx36 and Cx45 protein on cryosections of the human retina. After application of antibodies to Cx36, a punctated staining pattern in the inner plexiform layer (IPL) and to some lower extent in the outer plexiform layer (OPL) was detected (figure 5A). Due to the limited quality of the post-mortem human retinal tissue it was not possible to analyze the sublaminar distribution of Cx36-specific signals in both plexiform layers. The staining pattern obtained after application of antibodies to Cx45 was less intense in the inner plexiform layer (IPL) but more prominent in the outer plexiform layer (OPL) (figure 5C). These also punctate signals were smaller and more faint, at least in the IPL, compared to the corresponding Cx36-immuno signals (figure 5A). At first glance, the overall abundance and distribution of the punctate immunopositive staining patterns representing Cx36 and Cx45 protein in the human retina are reminiscent to the corresponding immunofluorescence signals detected in the mouse retina [25,28].

## Conclusion

In former years research on gap junction mediated signal transmission and its modulation in the mammalian retina focused on three aspects: (i) Coupling of photoreceptor cells and the improvement of signal-to-noise ratio under various light conditions. (ii) Horizontal cell coupling with respect to lateral inhibition and (iii) AII-ON-cone bipolar cell coupling transferring rod-mediated signals into the cone pathway under mesopic light conditions [42,43].

Most experiments were done in lower vertebrates like fish, frog, turtle or mudpuppy [6,31] but also results from higher vertebrates like cat, rabbit, mouse and rat contributed to the identification of either homologuous or heterologuous gap junction coupling between various retinal neurons [16,23,44].

In the recent years, research was focused on gap junction-mediated coupling between retinal neurons in mice, since the targeted deletion of connexin coding sequences after replacement with an appropriate reporter gene did not only help to unequivocally confirm cell-type specific expression of a distinct connexin in the mouse retina but also the physiological consequences after loss of this connexin [45]. Independent deletion of both the Cx36 coding region [28] and the Cx45 coding region [26] resulted in a reduction of the b-wave, thus implying that electrical synapses occurring between AII-amacrine and ON-cone bipolar cells are assembled by connexons heterotypically composed of Cx36 and Cx45 [5].

However, much less is known concerning the identity and distribution of connexin genes in the primate retina. It was shown that neighboring red and green cones in retinae of macaque monkeys are homogenously as well as heterogenously coupled by non rectifying gap junctions. Apart from these, blue cones seem to be electrically isolated, although they are homogenously coupled [32]. This indiscriminate coupling might cause a modest decrease in color discrimination that likely leads to the benefit of an increased luminance detection. Using a measured average junctional conductance of 650 pS, the authors further estimated 43 hypothetically open Cx36-channels per cone-cone junction assuming that the single-channel conductance of Cx36 does not exceed 15pS [32]. Until now it is not clear, if the same connexins that have been identified in the mouse retina are also expressed in the human or at least primate retina.

Therefore, we have started to investigate expression of those connexin genes known to be expressed in mouse retina in human retina. Human connexin36 transcripts were detected in total RNA prepared from human retina and the optical nerve after Northern blot hybridization. Furthermore, after application of Cx36-specific antibodies [25], Cx36 immunoreactivity was found from the inner plexiform layer (IPL) up to the ganglion cell layer (GCL) and also separately in the outer plexiform layer (OPL). Despite the limited quality of the post-mortem human retinal tissue, the staining pattern is partially reminiscent to that one detected in mouse retina, although intensity and the belt-like array pattern of punctate signals were different [25]. It seems likely that hCx36 protein mainly occurs in the IPL, similar to rodent nocturnal species [22]. A better quality of human retinal biopsy is needed to address the following question in further studies: Is there any accumulation of Cx36 protein in the inner part (ON sublamina) of the IPL, which participates in the rod pathway [46], the specialized circuit for night (scotopic) vision or in the outer part (OFF sublamina), like in the chick cone-dominated retina [47], that might suggests the independence of phylogenetic proximity and functional micro-wiring in retinal circuits.

Human Cx45 transcripts instead were identified in total human RNA from retina and iris diaphragm but not, as expected, in lens and optical nerve after Northern blot hybridization. Immunofluorescence analyses using Cx45-specific antibodies [25] yielded large and clear punctate signals in the OPL that were hardly detectable in the IPL and GCL. This is different from results observed in both rodent [25,31,56] and avian retina [48], where Cx45 was detected in different cell types, but mainly in the inner retina (IPL and GCL). In future studies a better morphological quality of post-mortem human retinal tissue might not only allow to verify these results but also to characterize, in combination with cell-type specific markers, the cellular distribution of hCx36 and hCx45 and other gap junction proteins, such as hCx43 [49] and pannexins [50] in the human retina.

Cx59 (GJA9) is restricted to human, since no orthologous connexin gene was found in the mouse genome [20]. A closer examination revealed the strongest phylogenetic relation to zebrafish Cx55.5 and a weaker relation to a group of connexins including mouse Cx57 (Gja10), porcine Cx60 and human Cx62 (GJA10). Sequence analysis of the chromosomal loci indicated that the mouse Cx57 gene (Gja10) on chromosome 4 and its surrounding is in perfect synteny to the human Cx62 gene (GJA10) on chromosome 6 coinciding with the high amino acid sequence identity (78%) of both genes [51]. Although partial sequences of mCx57 (Gja10) and hCx59 (GJA9) also represent relatively high sequence identities, their chromosomal location on human chromosome 1 and mouse chromosome 4 show no synteny. We therefore hypothesize that hCx59 (GJA9) might either be generated in the human genome by a gene duplication [52] and shuffling of genomic fragments related to hCx62 (GJA10) or that the orthologuous counterpart of hCx59 (GJA9) may have been lost through chromosomal breakage in the mouse genome. Most interestingly, transcription of hCx59 could only be detected in RNA from human testes after Northern blot hybridization [51], whereas RT-PCR amplicons confirmed at least weak expression of hCx59 in human retina.

Human connexin62 (GJA10) is apparently the true ortholog to mouse Cx57 (Gja10), since the genomes of both species have been completely sequenced and no other orthologuous gene pair shows higher sequence similarity [51]. Therefore it was interesting to explore if both connexin genes also share similar gene structures. We aligned the short coding sequence of mouse connexion57 exon 3 [30] to the genomic fragment containing the human connexin62 gene (GJA10) and identified the same 12 amino acid residues coding for mouse exon3. Although the intron between exon 2 and exon 3 of human Cx62 is about 25 kb long and thus much more extended than the corresponding intron (4 kb) of mouse connexin57 [30], it is functionally spliced in human retina. Additionally, transcription of the human Cx62 gene (GJA10) could also be detected after Northern blot hybridization only in human retina. Regarding the corresponding gene structure and the retina-restricted transcription, it is tempting to speculate that human Cx62 (GJA10) may be expressed in human horizontal cells similar to the expression of mouse connexin57 (Gja10) in mouse horizontal cells [30]. Expression of Cx57 could also be demonstrated in A-type horizontal cells of the rabbit retina [53]. Therefore, human Cx62 protein may contribute to connexin hemichannels at the tips of horizontal cell dendrites, which have been suggested to mediate negative feedback from human horizontal cells to cones [54].

In further investigations, the characterization of specific antibodies directed against human Cx59 and Cx62 proteins would help to clarify their cell-type specific expression.

## Abbreviations

CX: connexin; AA: amino acid residues; NT: nucleotides; RT-PCR: reverse transcriptase polymerase chain reaction; ORF: open reading frame; EST: expressed sequence tag; B-WAVE: activity of retinal interneurons; OPL: outer plexiform layer; IPL: inner plexiform layer; ONL: outer nuclear layer; INL: inner nuclear layer.

## Competing interests

The authors declare that they have no competing interests.

## Authors' contributions

GS carried out sequence analyses, Northern blot hybridizations, RT-PCR analyses and the immunofluorescence studies. Additionally he drafted the manuscript.

AJ participated in the design of the study and revised it critically and contributed to the human eye biopsies after organ donation including the informed consent.

NK performed the tissue preparation and contributed to the human eye biopsies after organ donation.

KW designed this study, and participated in the coordination and critical reading of the manuscript.

All authors read and approved the final manuscript.

## Pre-publication history

The pre-publication history for this paper can be accessed here:

http://www.biomedcentral.com/1471-2415/10/27/prepub
